# Awareness and the Arguments for and against the International Classification of Functioning, Disability and Health among Representatives of Disability Organisations 

**DOI:** 10.3390/ijerph120303293

**Published:** 2015-03-18

**Authors:** Jörgen Lundälv, Marie Törnbom, Per-Olof Larsson, Katharina S. Sunnerhagen

**Affiliations:** 1 Department of Social Work, University of Gothenburg, Box 720, 405 30 Gothenburg, Sweden; E-Mails: Marie.Tornbom@socwork.gu.se (M.T.); Per-Olof.Larsson@socwork.gu.se (P.-O.L.); 2 Department of Surgical and Perioperative Science, Umeå University, 901 85 Umeå, Sweden; 3 Research Group for Rehabilitation Medicine, Institute of Neuroscience and Physiology, Sahlgrenska Academy, University of Gothenburg, Per Dubbsgatan 14, 413 45 Gothenburg, Sweden; E-Mails: Marie.Tornbom@neuro.gu.se (M.T.); Katharina.Sunnerhagen@neuro.gu.se (K.S.S.)

**Keywords:** International Classification of Functioning, Disability and Health (ICF), disability organisations, representatives, awareness, social policy

## Abstract

Disability organisations have not been engaged in the debate about the International Classification of Functioning, Disability and Health (ICF) in Sweden. We wanted to know representatives’ attitudes about the ICF. The aim of the study was to elucidate the arguments for and against the ICF among representatives of disability organisations. The study consisted of eighteen representatives (from six disability organisations) that answered electronic questionnaires. The questionnaires involved ten open questions about the arguments for and against the ICF. The answers of the questionnaires have been categorized according to qualitative content analysis. Our results indicated four themes: awareness, arguments for and against, influence and the future. More than half of the representatives had very limited awareness of the ICF. There was an explicit criticism of individual classification but more positive comments about classification on an aggregated level. The most important issue for representatives was influencing social policy in society, not learning and spreading information about the ICF.

## 1. Introduction

In 2001, the International Classification of Functioning, Disability and Health (ICF) was approved by the World Health Organisation [1]. The intentions of the ICF were to create a classification system based on integration between the social and medical model of disability. This classification has created a scientific platform in order to form a mutual language globally to describe health-related issues between medical professionals and people with disabilities. The intentions were also to compare data from different countries [1]. The contents of the ICF have been used as a tool for statistics, research, clinical practice, social policy and education [2]. Thousands of scientists from all over the world, though predominantly from Europe and North America, have been engaged in producing conceptual papers or papers reporting the development of the ICF and of ICF-related instruments during the time period 2001 to 2009 [3].

The development of the ICF was also based on input from disability organisation s in different countries, who have acted on and made comments about the design and content. Rachel Hurst, engaged in civil rights for people with disabilities, wrote that the ICF made it possible to adopt an interactive model instead of focusing on a medical model of disability and to integrate environmental factors [4]. The risk of medicalization, dehumanization and increased classification of persons, with the use of the ICF has caused concern among scientists and other professions in the disability field [5].

Mark Priestley *et al.* highlighted the need for disability organisations to actively work with scientists to develop and encourage disability research [6]. The cooperation between disability organisation s and researchers has recently been identified as a priority in Europe [6,7]. The concept of handicap was removed from the classification because it only focused on the physical dimension of disability and therefore participation restriction was introduced.

The concept of disability has been fully adopted by the ICF, a concept with a bio-psychosocial dimension [4]. Disability organisations within Sweden (e.g., the Swedish Association of People with Mobility Impairments (Diversity Human Rights—DHR) and the Swedish Disability Federation (HSO)) have made little comment on the ICF since its launch in Sweden. As far as we know, earlier studies have not investigated the awareness that disability organisations have of the ICF. Therefore, we wanted to focus on representatives’ awareness and arguments for and against the ICF in Swedish disability organisations.

## 2. Methods

The study is empirical and based on the information from ten women and eight men who represented six prominent and well-known disability organisations at regional and national levels in Sweden. The selection of organisation was a conscious one and we turned to organisations that predominantly assist persons with physical disabilities and to an umbrella organisation that has a broad coordination function. The conscious choice of organisations was made by sending the questionnaires to people who working within different disability organisations in different parts of Sweden. The individuals selected worked at different levels within the organisations and their focus allowed them to politically influence disability policy at the local, regional and national level. Also included were larger organisations that focus on physical disabilities.

All participants received the questionnaires with ten open questions electronically via e-mail, and they were asked to respond in writing (Appendix). The questionnaires included questions about awareness and arguments for and against the ICF within disability organisations in 2001 and 2010. We wanted the participants to compare the arguments for and against the classification system over the course of a decade within their organisation s. Furthermore, the representatives were asked if their disability organisations had spread knowledge about the ICF in society and what was their opinion of the use of ICF going forward.

The representatives were asked to give their views of the pros and cons of the ICF. Two of the representatives were also interviewed via telephone. Data collection was performed during 2010–2012. The answers were categorized with qualitative content analysis. We have used a qualitative contents analysis methodology. We categorised all answers that came from participants in the study. The two researchers who analysed and categorised the answers did so independently of one another.

## 3. Results

### 3.1. Awareness of the ICF

More than half of the representatives had limited awareness of the ICF and answered briefly. Some said they had never heard of the ICF, or they had not heard discussion about the ICF either nationally or internationally. One informant states “Rather unkown. And no one is working to get it known, is there?” Another informant tells: “I believe there are a few users/patients that are aware of the ICF, I believe the information about the ICF is inadequate”.

Some of the representatives that knew about the ICF indicated that they felt they knew less about the ICF in 2001 as compared to 2010. Some said that the intention of the ICF was to combine the medical and social aspects of disability, and one respondent remembered that there were discussions about whether the ICF had stronger medical implications than social. The respondent indicated that he/she was concerned that the social implications had received less attention than the medical implications. One representative indicated that even though they liked the ICF, they were aware that several organisations argued it was hard to understand and use. For several representatives, the ICF was deemed not worth talking or learning about and was considered a non-issue. One representative said that if the ICF is only used by a few people, then the benefit is questionable. Since most of the disability organisations had very little knowledge of the ICF, they had not been actively spreading information about the ICF. 

### 3.2. Arguments For and Against

The core of the argument against the ICF consists of empowerment *versus* the fear of professional’s authority over the disabled individual.

The most explicit argument against the ICF was a criticism about classification and that there have never been arguments for this type of individual classification within disability organisations. There was concern that the ICF, categorizing people with disabilities, could be misused by medical and social professionals (e.g., medical doctors, occupational therapists, physical therapists and social workers), politicians, authorities and scientists. This misuse could lead to the marginalization of people with disabilities, and several representatives considered individual classifications to have no value.

In their answer, a participant explained their criticism of the ICF: “We (the organisation) were already sceptical of the ICF long before it was launched here in Sweden. We thought that the detailed code system objectified people in an unpleasant way and it feels foreign/strange from a Swedish perspective. We don’t, as many other countries do, systematically put people with disabilities into categories or grades—and we don’t want to see that happen either. Unfortunately it would seem that professionals have fallen in love with the ICF and we fear that its use will only increase”.

Some respondents indicated that the ICF concentrates on the individuals, thereby making people objects, and all the details that some authorities and health organisations investigate according to the ICF could be offensive to users.

A participant answered: “I consider that the ICF would become a classification system that objectifies people with disabilities. Also that the ICF in all its rich details was degrading/offensive”. Another participant in the study described the experience of being offended: “Many members who have been victims of assessment criteria have felt offended by the questions asked. Certain questions are irrelevant to the information that was sought. The interviewer “slavishly” followed the classification system and as a result asked all the questions that are contained within the ICF, disregarding the fact that not all of them are relevant. This was experienced as very offensive”.

The questions asked by professionals were sometimes not considered necessary or even relevant; the classification system has often been used as an instrument. However, one respondent considered the detailed individual classification that was criticized above, to be relevant.

Those who were more positive about the ICF found different elements that could be used to improve life conditions of people with a disability. One representative indicated that the knowledge could be used to reduce challenges and increase the quality of life for people with a disability. A participant explains: “personally I think that the ICF is an important tool for all categories of staff who work with people with different disabilities. It is available at a detailed level and can be translated into practical work to improve and enhance the quality of life for people with disabilities”.

Other representatives argued that with the ICF, factors about the surroundings emerged, often earlier forgotten and the ICF focused on what hindered and what alleviated life conditions. Some argued that the differences between impairment, disability, activity and participation were described thanks to ICF and a structure was created. One informant tells about the importance that ICF can be employed by all professionals in a team. The informant says: “the ICF model is good as a comparison tool but is rather difficult to learn how to apply in a multiprofessional team. Many different professional groups’ assessment ate intertwined”. Another informant described it as follows: “The discussion (regarding the ICF) focused on uniting the medical and the social aspect. Depending on which “camp” you came from, some were against the feeling that ICF had a strong medical emphasis and others argued still there was too little focus on social and environmental factors. In the beginning and still ongoing, there are discussions about where the boundary between activity and participation are”.

One responded that there also was a need for a development of the earlier International Classification of Impairments, Disabilities and Handicaps (ICIDH) system. In one organisation, they had no arguments against the ICF in 2001 and thought that comparing studies both nationally and internationally could be performed thanks to the aggregated results of the ICF.

One person expressed a positive view of the ICF in their answer: “I believe and hope that we can do comparison studies that can be used to develop healthcare in the future that benefit us all”. The same organisation indicated that even invisible individual conditions could become visible thanks to the ICF. One positive argument about the ICF was that the ICF does not focus on diagnoses.

### 3.3. Influence and the Future

Some representatives wrote there was an obvious lack of focus on social policy within the ICF and that was the most important issue for disability organisations. Therefore, the representatives thought there was no point learning more about the ICF. There was also a criticism that they experienced ICIDH and the ICF as emerging without opinions from disability organisations.

The criticism from several organisations in Sweden is about not having been involved in the process of introducing the ICF. According to the respondents, only researchers and the professionals use the ICF as a tool to identify needs for support. For disability organisations the ICF is of limited value. One respondent argued that disability organisations are struggling to survive; they have no resources to work with and spread information about the ICF. A minor disability organisation has no possibility to spread information about the ICF. Informants stated that without the knowledge of the ICF, its content and use, it would be difficult for the classification to survive.

Relevant knowledge has to be spread. People using the ICF and scientists must take responsibility to spread information about the ICF. Even in the future, some argued, professionals might be more interested in the ICF than disability organisations. One informant wrote that research based on the ICF could be relevant to the development of hospital care and municipal care. The informant says: “Hard to say, but I believe and hope that we can do comparative studies that may be of general benefit when developing health care and community care in the future”. Another person states that there are possibilities to spread the knowledge of ICF in society “The possibility it great, provided that the information is spread, gets disseminated to all interested groups”.

The world needs a common language; the ICF could be developed after being inspired by disability organisations. One thought that disability organisations should focus on the abilities of the ICF and not the difficulties. One respondent suggested that the concept of participation should be the base for evaluation.

## 4. Discussion

Despite turning to key individuals within the representative organisations, we were surprised by the lack of knowledge of the ICF, and in certain cases also by the arguments for and against the ICF. We had to be satisfied with categorising those answers we received, despite our surprise.

Our reasons for wanting to bring this information to light is to demonstrate how far apart the persons with disability are from the professionals who use the ICF in both their understanding and opinion of the ICF. This study described and explored the awareness and arguments for and against the ICF among eighteen representatives within six disability organisations. Our study indicated that only a few respondents had thorough knowledge and very good understanding of the ICF.

We are surprised that more than half of the representatives were unaware of the ICF. However, the respondents had opinions, and the opinions were categorized. One possible conclusion from this study is that the ICF—as a phenomenon—has become better known among scientists and medical and social professionals than among representatives of disability organisations. According to this study, there seems to be a wide gap between medical and social professionals’, scientists’ and members of disability organisation’s awareness of the ICF. One person argued that scientists and professionals should be responsible for involving disability organisations throughout scientific projects of disability research. Our results show that the organisations want to participate, as suggested by Priestley *et al.* [6].

The most explicit result was that the respondents were reluctant to use the ICF-classification on an individual level in clinical practice. Patients have been offended when asked questions by medical professionals according to the ICF-classification systems. When following that classification system, the professionals’ focus could be misdirected away from their patients’ individual needs. In a study of implementation of the ICF, the authors argued for global standardized implementation of the ICF in clinical rehabilitation [8]. We argue that the patients’ own experiences and individual needs should be the professional focus.

Another result told us that some representatives did not trust the ICF as a social policy instrument. In Sweden there was an ambition when the ICF was implemented that it might be used as a social policy instrument. Today, the ICF is a model that that is used by the Swedish National Board of Health and Welfare and the Swedish medical staff are asked in the charts to describe not only signs and symptoms of disease. Physicians in Sweden must also complete the activity limitations in sickness certificates for the Swedish National Insurance Board (Försäkringskassan). One Swedish study shows that only one third of the physicians have completed such data [9]. The result in our study confirmed previous worries about how detailed classification might be used in a negative way [4]. In contrast, the ambitions from scientists and professionals seem to be that the ICF on an aggregated level might influence policy-makers in fulfilling obligations under the UN Convention for persons with disability [10,11].

One limitation of this study was that a limited number of participants were involved. A second limitation with our study might be that our questions might have been guiding since we asked twice regarding arguments against ICF but once concerning pros. We argue that more research is needed to reduce the risks that the ICF will be misused and, above all, to increase the involvement of disability organisations globally.

## 5. Conclusions

There is a need to work closer with the disability organisations in Sweden (and perhaps elsewhere) if an understanding and acceptance of the ICF will occur in them. To develop the ICF, a partnership is needed.

## Appendix

### Questionnaire:

The questions used in our electronic form were as follows:

1. In your opinion, what arguments are there within the international handicap/disability movement for and against the ICF, as at **2001**?
2. In your opinion, what arguments are there within the international handicap/disability movement for and against the ICF **today**?
3. In your opinion, what arguments were there within your organisation for and against the ICF in **2001**?
4. In your opinion, what arguments are there within your organisation for and against the ICF **today**?
5. What do you see as arguments against the ICF **today**?
6. How do you feel the ICF is known among professionals today? Among disability organisations? Among disabled persons/patients?
7. Does the handicap/disability movement have an understanding of the ICF and is that understanding correct?
8. How do you feel that the international handicap/disability movement has informed and spread knowledge of the ICF within society?
9. How do you feel that your organisation has informed and spread knowledge of the ICF with its members and others within society?
10. What meaning do you think that the ICF will have **in the future**?
